# Zinc toxicity stimulates microbial production of extracellular polymers in a copiotrophic acid soil

**DOI:** 10.1016/j.ibiod.2016.10.004

**Published:** 2017-04

**Authors:** Marc Redmile-Gordon, Lin Chen

**Affiliations:** a Department of Sustainable Soils and Grassland Systems, Rothamsted Research, Harpenden, Herts AL5 2JQ, UK; b State Key Laboratory of Soil and Sustainable Agriculture, Institute of Soil Science, Chinese Academy of Sciences, Nanjing 210008, China; c Institute of Soil and Water Resources and Environmental Science, Zhejiang University, Hangzhou 310058, China

**Keywords:** Extracellular polymeric substances, Biodiesel co-product, Soil biofilms, Bioremediation, Heavy metal, Exudate polysaccharide, EPS, Extracellular polymeric substances, SMP, Soluble microbial products, BCP, Biodiesel co-product, CER, Cation exchange resin, SOC, Soil organic carbon, iLUC, Indirect land-use change

## Abstract

The production of extracellular polymeric substances (EPS) is crucial for biofilm structure, microbial nutrition and proximal stability of habitat in a variety of environments. However, the production patterns of microbial EPS in soils as affected by heavy metal contamination remain uncertain. Here we investigate the extracellular response of the native microbial biomass in a grassland soil treated with refined glycerol or crude unrefined biodiesel co-product (BCP) with and without ZnCl_2_. We extracted microbial EPS and more readily soluble microbial products (SMP), and quantified total polysaccharide, uronic acid, and protein content in these respective extracts. Organic addition, especially BCP, significantly stimulated the production of EPS-polysaccharide and protein but had no impact on EPS-uronic acids, while in the SMP-fraction, polysaccharides and uronic acids were both significantly increased. In response to the inclusion of Zn^2+^, both EPS- and SMP-polysaccharides increased. This implies firstly that a tolerance mechanism of soil microorganisms against Zn^2+^ toxicity exists through the stimulation of SMP and EPS production, and secondly that co-products of biofuel industries may have value-added use in bioremediation efforts to support *in-situ* production of microbial biopolymers. Microbial films and mobile polymers are likely to impact a range of soil properties. The recent focus on EPS research in soils is anticipated to help contribute an improved understanding of biofilm dynamics in other complex systems - such as continuously operated bioreactors.

## Highlights

• Biodiesel co-product (BCP) is demonstrated to support microbial EPS production in acid soil.
• Contamination of BCP treated soil with zinc (ZnCl_2_) increased the production of EPS polysaccharides.
• This increase in EPS was accompanied by an increase in the production of soluble uronic acids.
• Understanding EPS and SMP dynamics in soils will assist design of engineered bioprocesses in soils.
• BCP-driven processes in soils and reactors should include Zn as an influential factor in biofilm formation.

## 1 Introduction

Extracellular polymeric substances (EPS) are complex high-molecular-weight mixtures of polymers synthesized by microbial cells. EPS play cardinal roles in nutrient acquisition (Laspidou and Rittmann, 2002), stabilization and protection of biofilm structure (Flemming and Wingender, 2010), microbial adhesion to the habitat matrix (Cammarota and Sant'Anna, 1998, Hong et al., 2013), and impart resistance to toxicity (Henriques and Love, 2007, Joshi et al., 2012). Indeed, stress seems to be a common factor underlying many of the triggers to production of EPS. These stressful triggers can include physical shear, bacteriophage abundance, organic contaminants, biocides and antibiotics (Vu et al., 2009). In the past few decades, the majority of reports on the production and roles of microbial EPS focus on aqueous environments, such as marine (Beech and Cheung, 1995, Zinkevich et al., 1996) and wastewater treatment systems (Sheng et al., 2010, Miao et al., 2015, Wei et al., 2016). Exogenous organic substrate and heavy metal ion concentration are crucial factors influencing microbial EPS production and biofilm formation in these systems. For instance, silver ions and nanoparticles affect the composition of phototrophic biofilm in operated bioreactors (González et al., 2015), and copper and iron concentrations affect the profile of phenolic compounds exuded by marine microalgae (Rico et al., 2013, Lopez et al., 2015).

Zinc (Zn) is a heavy metal of particular concern. Zn^2+^ can be highly damaging for the environment and organisms exposed to it, and is routinely discharged during anthropogenic activity in the mining, chemical, pulp and paper industries (Lu and Chiu, 2006). A recent study of wastewater treatment systems investigated the biological complexation of Zn^2+^ by EPS and found that stretching vibration of O—H, N—H groups and C=O bonds were implicated (Wei et al., 2016). Elsewhere, in agricultural technology, the Zn-tolerant plant-pathogen (*Xylella fastidiosa*) was shown to produce large amounts of EPS-polysaccharide in response to additions of Zn^2+^ to flow cells (Navarrete and De La Fuente, 2014). Indeed there is a growing interest in the responses and roles of microbial exudates in porous media, especially soils, for the purposes of bioengineering and agronomy (e.g. Or et al., 2007) but investigations of native and community-wide EPS responses in soils directly are rare (e.g. Redmile-Gordon et al., 2014b). While easily accessible/labile C is now understood to be a pre-requisite for substantial production of EPS from soil biota (Nunan et al., 2003, Redmile-Gordon et al., 2015a), the influence of any heavy metal contamination on proteinaceous and polysaccharide exudate production by soil native microbial populations *in situ* has not yet been reported.

Besides the knowledge-gap in soil systems, the practical significance of EPS in other environments as a tolerance mechanism against heavy metal contamination suggests there may be further un-explored value in the understanding of soil EPS dynamics such as for more efficient bioremediation of contaminated soils. EPS is also thought to be vital for restoring a range of other important soil ecological and agronomic functions that are related to altered hydraulic dynamics and soil structure (Or et al., 2007). Recently proposed methods to measure EPS in soil (adapted from methods used in aquatic sciences) were applied by Redmile-Gordon et al. (2015a). The authors used ^15^N isotope probing -and measures of soil ATP- to demonstrate that extraction with cation exchange resin (CER) could be used to contrast changes in total polysaccharide and protein fractions exuded by the native soil microbial biomass. This approach builds upon one of the most frequently used ways to extract EPS in saturated aqueous systems (Frolund et al., 1996). Through the application of this method, biodiesel co-product (BCP) was subsequently shown to be an efficient and sustainable choice of substrate to support EPS production in soil (Redmile-Gordon et al., 2015b). BCP was selected as a C substrate to support microbial metabolism owing to a global and pressing need to reconcile issues of food security and bioenergy through integrated synergies (Redmile-Gordon et al., 2015b, Kline et al., 2016). The growing range of uses for BCP in soils ranges from the capacity to reduce direct N_2_O emissions (Alotaibi and Schoenau, 2013) preventing NO_3_^−^ contamination of groundwater (Redmile-Gordon et al., 2014a) and supporting production of EPS via the native soil microbial biomass (Redmile-Gordon et al., 2015b).

Here, we present a laboratory experiment to investigate the responses of microbial EPS production to BCP in a soil contaminated (and not) with Zn^2+^. The objective of this study was to quantitatively determine how polysaccharide and protein exudate fractions of a heterotrophic soil microbial biomass (utilising BCP as a substrate) was affected by Zn stress. A further objective was to categorise these responses broadly as either belonging to the highly soluble fraction (SMP) or the relatively insoluble but CER extractable fraction: EPS.

## 2 Materials and methods

### 2.1 Soil sampling and experimental design

Samples of sandy soil (8.0% clay, a Cambic Arenosol, FAO classification) were collected from the surface horizon (0–23 cm) of a permanent grassland area adjoining plots of the ‘Market Garden Experiment’ at Rothamsted Experimental Farm (51°59′ N, 0°35′ W), Husborne Crawley, Bedfordshire, UK. The soil contained 16.86 mg g^−1^ organic carbon (SOC), 1.55 mg g^−1^ total nitrogen (N) and a pH of 5.95 (pH as a suspension in boiled, cooled de-ionised water with soil:solution ratio 1:2.5). Twelve moist portions of soil (100 g oven-dry weight equivalent) were placed in glass funnels. These were arranged randomly to compare four treatments with three replicates of each. The treatments compared were: glycerol addition, biodiesel co-product (BCP) addition, BCP plus ZnCl_2_ addition, and no C addition (control). The Glycerol-C and BCP-C were provided at the start of the experiment at rates of 20 mg C g^−1^ soil. To ensure that the growth of native microbes and EPS throughout the soil were not limited by nutrient availability, ammonium nitrate and monoammonium phosphate were added to all soils at concentrations of 1.50 mg N g^−1^ soil and 0.35 mg P g^−1^ soil, respectively, other nutrients (K^+^, Ca^2+^, Mg^2+^, SO_4_^2−^, Na^+^ and Cl^−^) were provided at a concentration of 0.10 mg g^−1^ soil.

After 24 h of C and nutrient addition, 20 mL of 0.01 M CaCl_2_ was applied to the soil surface in each funnel. This step was repeated each day thereafter to simultaneously re-moisten the soil, remove excess substrate C from soil pores, and redistribute solutes as would occur in a more natural system exposed to weather in an open environment (Redmile-Gordon et al., 2015a). Dilute CaCl_2_ is commonly used in preference to deionized water in soil laboratory studies as a surrogate for rainwater owing to osmotic similarity (Jalali and Rowell, 2003). A contrasting three portions of 0.01 M CaCl_2_ were spiked with ZnCl_2_ to deliver 300 μg Zn^2+^ g^−1^ soil. These were added to three replicates of the BCP-amended soils each day and allowed to drain freely. This daily addition of Zn^2+^ is similar to the difference in Zn concentration between contaminated and uncontaminated soils taken from the same site described previously by Chander and Brookes (1991) with ‘uncontaminated’ soil yielding a concentration of 107 μg Zn g^−1^ soil by digestion (4:1 (v/v) HCl:HNO_3_).

All treatments were incubated in the dark at 25 °C for 7 days. While 10 days has previously been given for development of EPS in these conditions (e.g. Redmile-Gordon et al., 2014b) 7 days was chosen in the present study for analytical convenience. Importantly, it is around this time that EPS responses are likely to be detectable because EPS production is typically greatest around the transition between ‘log’ and ‘stationary’ growth phases (Wagner et al., 2003). From our previous observations of inflection points for cumulative CO_2_ release curves this point appears to occur sometime between 4 and 12 days in the conditions specified above. At the end of the 7 day incubation period, excess pore-water was removed by applying a 40 cm of mercury-equivalent vacuum to the funnel-outlet and the mesocosms were destructively sampled.

### 2.2 SMP and EPS extraction, quantification, and statistical analysis

The SMP and EPS extraction protocols were followed as described in the open access article by Redmile-Gordon et al. (2014b). Accordingly, the residual SMP fraction was extracted from moist subsamples (2.5 g dry weight equivalent) placed in 50 mL polypropylene centrifuge tubes (manufactured by Greiner) on an end-end shaker set to 2 cycles per second at 4 °C using 0.01 M CaCl_2_ at a 1:10 soil:solution ratio. Extracts were then centrifuged at 3200×*g* for 30 min, the SMP solution was decanted and frozen for subsequent analyses. EPS was then extracted from the remaining pellet by re-suspending in new tubes containing 25 mL of EPS extraction buffer. Buffer was prepared in 18 MΩ H_2_O to: 2 mM Na_3_PO_4_·12H_2_O (0.760 g L^−1^), 4 mM NaH_2_PO_4_·H_2_O (0.552 g L^−1^), 9 mM NaCl (0.526 g L^−1^), 1 mM KCl (0.0746 g L^−1^), adjusted to pH 7 with 1 M HCl and cooled to 4 °C. Sufficient CER (Dowex ‘Marathon C’ sodium form, strongly acidic, 20–50 mesh) was prewashed twice in the above buffer and then added as an amount equal to 178 mg per mg organic carbon in the untreated soil (Redmile-Gordon et al., 2014b), therefore, 7.50 g CER per 2.5 g soil sample in the present case. This was shaken at the same speed as for SMP removal but for 2 h at 4 °C. Samples were then centrifuged at 4000×*g* for 30 min and the supernatant transferred into new tubes. These were frozen and stored at −20 °C prior to analysis. Total polysaccharide and uronic acids were quantified as described by DuBois et al. (1956) and Mojica et al. (2007), respectively. The extracted protein content accounting for colorimetric interference from humified organic material in the extracts was measured using the Lowry technique as modified for microplate format and described in the open access article by Redmile-Gordon et al. (2013) except that no dilution of extracts were required, i.e. 100 μL of EPS extract was analysed by direct comparison against absorbance of one set of standards containing 0–100 μg Bovine Serum Albumin (Sigma A7906) mL^−1^ EPS buffer (described above). For the SMP extracts, the standard concentration range was identical except made in a matrix of 0.01 M CaCl_2_. All data were normally distributed, meeting the required assumptions for a one-way ANOVA without transformation. The LSD test was subsequently applied for comparison of means at a 0.05 significance level using GenStat (version 15).

## 3 Results and discussion

### 3.1 Value-added use of BCP as substrate for heterotrophic production of EPS

**Fig. 1:**
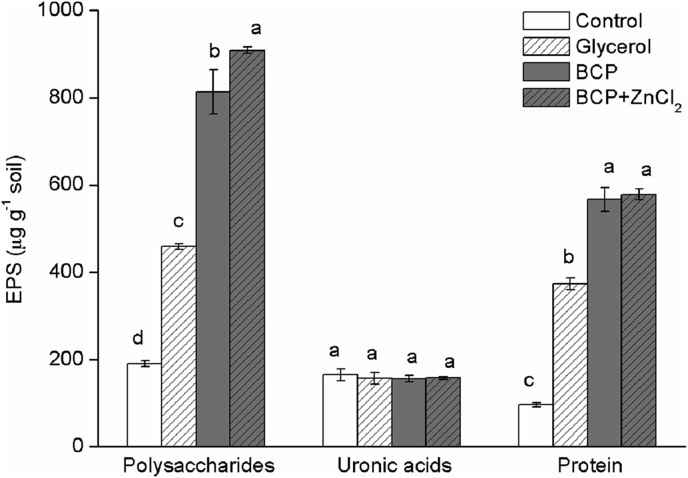
The concentrations of EPS indicators-polysaccharides, uronic acids, and protein in the control, glycerol, BCP, BCP plus ZnCl_2_ treatments. Error bars represent standard errors of three replicates. Different letters indicate that the mean differences are significant at the 0.05 level.

We found the concentrations of both EPS-polysaccharide and EPS-protein were significantly increased with the addition of organic C, especially through unrefined BCP addition (Fig. 1). The same trend for an increased EPS response to BCP vs. refined glycerol, except in a clay-loam soil of neutral pH was seen previously by Redmile-Gordon et al. (2015b). The aforementioned study also found that more EPS was produced from BCP made with recycled cooking oils compared to either refined glycerol or BCP produced from virgin oilseed rape. This is an important consideration in the selection of C substrates to support microbial processes either in soils or in bioreactors, as this enhances the wider benefits of making biofuels from waste oils. In the above case, the increase in EPS production was not due to any heavy metal content in the BCP, as heavy metal concentrations in all the C substrates applied were consistently below the metal content of straw biomass harvested from a reference stadard of pristine grassland (Parkgrass Experiment, Rothamsted Research, Harpenden, UK). In batch culture conditions, Freitas et al. (2010) also observed greater increases in EPS production from unrefined co-products of biodiesel when compared to pure glycerol. While the reasons for this are currently unknown, glycerol is a completely hydrophilic C source, whereas BCP also comprises salts of fatty acids, and small quantities of hydrophobic fatty acid methyl esters, and unreacted mono- and di-glycerides (Zhou and Boocock, 2006). The uses of organic materials (e.g. biochar and organic composts) in the remediation of Zn-contaminated acid soils or sites have been widely reported (e.g. Soares et al., 2015, Chiang et al., 2016, Shen et al., 2016). However, BCP shows additional potential in remediation technology through augmenting the production of microbial EPS and SMP. Importantly, the production of BCP requires less energy than refining it to extract components such as high purity glycerol but refining is still routinely performed in higher tech biodiesel plants. Direct uses for BCP in remediation would negate the need for expensive biodiesel-plant machinery and thus improve the feasibility of energy security projects for community-scale biodiesel enterprises in developing countries (Raghareutai et al., 2010). On the larger scale, international policy for biofuels is negatively affected by notion that biofuels are the driver of indirect land-use change (iLUC). iLUC is a genuine problem threatening dwindling wildlands especially in developing countries (Baral and Malins, 2016). While the logic behind resting blame for iLUC with biofuels is questionable (Kline et al., 2016) the use of BCP as a soil improver in marginal areas could decrease land-use pressure on already high-functioning soils which would be a quantifiable reversal of iLUC (Power, 2010, Sanchez et al., 2012, Redmile-Gordon et al., 2015b). The use of BCP to support microbial growth and EPS production in marginal soils is therefore of particular interest for sustainable intensification.

### 3.2 Zn contamination of copiotrophic soils: microbial biomass responses and implications

Evidence pointing towards EPS as a microbial tolerance mechanism against Zn stress is more established in non-soil disciplines such as rivers and clean, simplified systems, with visualisation techniques in soils being almost impossible due to the plethora of densely packed opaque minerals and decaying organic materials (Hao et al., 2016). Extraction approaches remain highly challenging due to the range of potentially interfering organics typical of most soils (Redmile-Gordon et al., 2014b). However, in the present study we show that the total EPS polysaccharide fraction increased in response to Zn^2+^ addition to a soil already containing a complex microbial community. These results are in accordance with, and expand upon, the environmental relevance of findings in other studies. For example, the increased cell-specific production rates of EPS-polysaccharides previously observed in flow-cells of monocultures spiked with Zn^2+^ (Navarrete and De La Fuente, 2014) appears to hold true, both a) in the highly complex soil environment and b) as a general dynamic in our soil at the scale of the microbial community. However, in the study of Wei et al. (2016) it was found that the measured EPS concentration had decreased after exposure to Zn. In this case the authors had used much higher concentrations of Zn, and EPS concentration (before and after) was estimated by application of hot NaCl. The application of hot extracts is known to cause heat shock, cytoplasm leakage and even lysis (Sun et al., 2012). Hot extraction of EPS is thus likely to co-extract internal cell biopolymers and so results should be interpreted with care: potentially reflecting the toxicity to microbial cells (and reduced biomass growth) as opposed to reflecting a genuine reduction in cell-specific production of EPS.

Previously in soil science, Zn has also been demonstrated to reduce the size of the soil microbial biomass (e.g. Renella et al., 2002) and inhibit nutrient turnover (Smolders et al., 2003). However, an increased CO_2_ evolution per unit of microbial biomass (increased ‘metabolic quotient’ or ‘qCO_2_’) typically accompanies this phenomenon: Chander and Brookes (1991) attributed this to “less efficient utilization of substrates for biomass synthesis” and thus implied a redirection of C towards tolerance mechanisms. Smolders et al. (2004) suggested that in locations where they found total cell biomass was not affected by availability of Zn that some unmeasured tolerance mechanisms must have existed to prevent the expected toxicity. In the study of Smolders et al. (2004), soils were also spiked using ZnCl_2_ and at concentrations up to 1000 μg Zn g^−1^ soil. Importantly, no studies are known to show an increase in microbial biomass due to contamination with Zn^2+^. The EPS production efficiencies in uncontaminated soils (also amended with glycerol and BCP) were previously found to depend on the C/N ratio of available substrates for microbial growth (Redmile-Gordon et al., 2015a). While the effect of Zn^2+^ addition on EPS production efficiency per se (μg EPS nmol^−1^ microbial ATP, or μg EPS nmol^−1^ biomass C) was not an objective of the present study, the addition of excess Zn^2+^ is highly unlikely to have increased the microbial biomass (again, as no soil studies have ever shown this), and so the clear increase in EPS and SMP production between soils given BCP and those given BCP plus Zn^2+^ points towards microbial allocation of metabolites into Zn tolerance mechanisms.

**Fig. 2:**
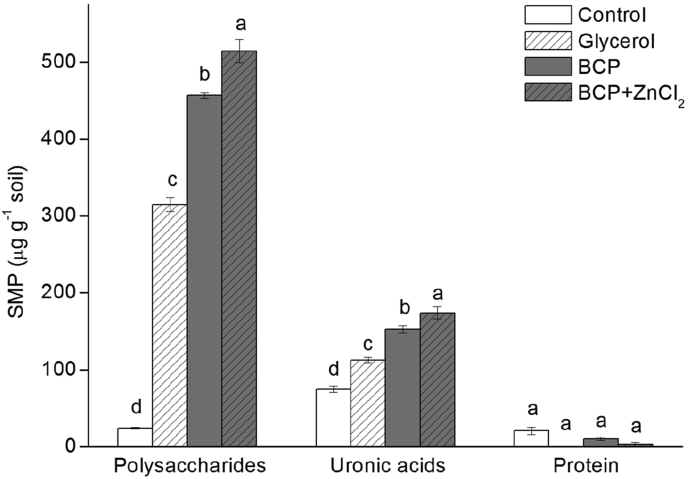
The concentrations of polysaccharides, uronic acids, and protein in SMP fraction in the control, glycerol, BCP, BCP plus ZnCl_2_ treatments. Error bars represent standard errors of three replicates. Different letters indicate significant differences at *P* < 0.05.

The increased SMP-uronic acid (Fig. 2) and EPS-polysaccharide production (Fig. 1) represent more, and less mobile fractions, respectively. These are therefore useful starting points from which to investigate the ecological significance of exudate dynamics, with practical potential for example in the bioremediation of Zn from acid soils. EPS are considered as a highly useful adsorbent for heavy metal contamination, owing to the many functional groups, including carboxyl, amine, and hydroxyl groups for example earning a role in treating metal-loaded wastewater (Guibaud et al., 2004, Zhang et al., 2006, Yin et al., 2011, Wei et al., 2016). However, spatial analyses in artificial systems (e.g. composed of Fe^2+^, Fe^3+^ and *Rhodobacter ferrooxidans*) have shown that Zn^2+^ exhibits greater affinity for cell surfaces than for glycoconjugates in the EPS (Hao et al., 2016) in such cases it follows that more mobile complexing components such as SMP would be required to enable translocation away from the cell. We found that uronic acid concentrations in the EPS (less mobile fraction of exudates) did not vary between any of the treatments (Fig. 1). Pereira et al. (2009) claimed that EPS uronic acids were mostly indicative of cyanobacterial activity, while in the present study, no light was provided, so cyanobacterial activity would have been negligible. Nonetheless, we found statistically significant increases in uronic acid content of SMP amounting to a 100% increase over control with the addition of BCP, which became an increase of more than 130% when Zn^2+^ was subsequently added (Fig. 2). This points towards *mobile* uronic acids being a more responsive component to Zn^2+^ than uronic moieties in the EPS. Kaplan et al. (1987) investigated Zn association with exudates of *Chlorella stigmatophora* and found that complexes were formed with concentration being directly proportional to the quantity of dissolved polysaccharides and speculated this was due to complexation by the mobile uronic acid fraction. Indeed, aqueous solutions of naturally produced uronic acids have since been used in bioremediation efforts to remove Zn and other heavy metals from contaminated soils (Fischer and Bipp, 2002). However, other work has also shown that the capacity of uronic moieties for Zn complexation can be low (e.g. Guibaud et al., 2004) with the efficacy of heavy metal sorption being dependent on concentrations of competing cations and oxidation status of the environment being studied (Hao et al., 2016). Further studies in diverse media such as soils therefore have much to offer in this regard.

The present study concerns the EPS production of a native soil microbial community exposed to Zn^2+^contamination in the laboratory. Additional studies in field-soils of more complex systems (including plants) are needed to determine if similar dynamics occur in the field. Meanwhile, laboratory studies of the mobility of Zn and other heavy metals both independently and as mixtures would be informative, including rates of exchange between soil solution, soil colloids, and the more mobile (SMP) and less mobile fraction of exudates (EPS). Currently the interplay between metals and biofilms in soils is still poorly understood which limits the efficacy of remediation technologies like phytoextraction (Sheoran et al., 2016). Indirect mechanisms of Zn transport in the extracellular habitat, not related to complexation of Zn have also been postulated to explain the removal of Zn from soil. For example, where inoculation of soil with EPS-producing *Pseudomonas* decreased the sorption of Zn to soil surfaces (Drozdova et al., 2014), here, the authors ascribed this effect to the shielding of active sites of soil organic matter with inert bacterial polysaccharides. The wealth of unknowns in this area raises many questions such as where and when do EPS and SMP fractions increase/decrease the bioavailability and mobility of heavy metals.

Finally, regarding the proteinaceous fraction of exudates, in the present study EPS-protein was enhanced by organic C addition, but not by inclusion of Zn^2+^ (Fig. 1). In the SMP fraction, protein was almost undetectable (Fig. 2). Our findings are in line with other reports proposing that enzymes are not released indiscriminately into solution as soluble biopolymers, but are instead retained by the EPS (Flemming and Wingender, 2002, Romani et al., 2008). While Tonietto et al. (2014) found that Zn had a strong affinity for proline and hydroxyproline residues, the apparent lack of any association between Zn and proteinaceous exudates in our data do not suggest that this dynamic holds true in this acid soil. As a consequence it might be interpreted that EPS-protein had little to do with any tolerance mechanism against Zn toxicity. However, it remains a possibility that a pool of non-extractable proteinaceous Zn complexes was accumulating in the soil. Future work tracking EPS dynamics with heavy metal fluxes into stabilized soil organic matter would be informative in this regard.

## 4 Conclusions

The data presented here indicate that the EPS differences between the BCP and BCP plus ZnCl_2_ treatments can be confidently ascribed to Zn^2+^, suggesting that in acid soils, native microbial communities are likely to tolerate heavy metal toxicity by two modes of action: i) stimulating production of EPS-polysaccharides, and ii) exuding soluble uronic acids. Given that altered EPS dynamics will affect the mobility of toxic metals via soil physical and biochemical changes, the further investigation of the movement and fate of Zn^2+^ as affected by EPS production could help better inform current practice and illuminate new opportunities for soil management and remediation. We therefore propose that measurements of soil microbial EPS and SMP are included in studies investigating the progress of remediation of soils that have been deleteriously exposed to heavy metals. As the soils dataset expands, we envisage that models able predict EPS responses to metals and the subsequent impacts of EPS on spatial and physical aspects of soil function (e.g. Or et al., 2007) will yield useful data for biotechnological application in non-soil environments. The method of EPS extraction from soil using CER exchange described here was adapted from -and is sufficiently similar to- existing approaches applied in wastewater treatment systems. Method similarity will ensure data obtained from soils is cross-relevant to other environments, and the advancement of technology in biotechnological applications such as continuously operated bioreactors (Sheng et al., 2010, Redmile-Gordon et al., 2014b). In this way, the outputs of each scientific discipline can synergistically contribute to the other, and to the sustainability of science generally: by magnifying returns, and deepening our understanding of the triggers to community-wide biofilm formation and dispersal.
